# Reverse of Acute and Chronic Morphine Tolerance by Lithocholic Acid *via* Down-Regulating UGT2B7

**DOI:** 10.3389/fphar.2016.00404

**Published:** 2016-11-01

**Authors:** Zizhao Yang, Li Li, Haihong Hu, Mingcheng Xu, Jingkai Gu, Zaijie Jim Wang, Lushan Yu, Su Zeng

**Affiliations:** ^1^Laboratory of Pharmaceutical Analysis and Drug Metabolism, Zhejiang Province Key Laboratory of Anti-Cancer Drug Research, College of Pharmaceutical Sciences, Zhejiang UniversityHangzhou, China; ^2^Department of Pharmacy, Zhejiang Hospital, Zhejiang Provincial Key Lab of GeriatricsHangzhou, China; ^3^Research Institute of Translational Medicine, School of Life Sciences, Jilin UniversityChangchun, China; ^4^Department of Biopharmaceutical Sciences and Cancer Center, University of Illinois at Chicago, ChicagoIL, USA

**Keywords:** lithocholic acid, UGT2B7, CaMKIIα, cAMP, morphine tolerance

## Abstract

Lithocholic acid (LCA) deposited in human livers always induces drastic pains which need analgesic drug, like morphine to release. Our research showed that LCA can effectively inhibit uridine 5’-diphospho-glucuronosyltransferase 2B7 (UGT2B7) in morphine tolerance-like human normal liver cells, HL-7702, then increase μ-opioid receptor (MOR) and calcium–calmodulin dependent protein kinase IIα (CaMKIIα) expression. *In vivo* assay, UGT2B7 was significantly repressed in the livers of acute or chronic morphine tolerance mice pretreated with LCA (10, 50, and 100 mg/kg, p.o.). To investigate the connections between LCA function performance and change of UGT2B7 enzymatic activity in mice livers, two morphine metabolites, morphine-3-glucuronide (M3G) and morphine-6-glucuronide (M6G) were quantified by solid phase extraction (SPE)–HPLC–MS/MS. The result indicated no matter in acute or chronic morphine tolerance, the concentrations of M3G and M6G were all decreased, the later one fell even more. Besides that, 50 mg/kg of LCA administration can prevent auto-phosphorylation of CaMKIIα at Thr286 in acute or chronic morphine tolerance mice prefrontal cortexes (mPFCs) due to synthesis increase of cyclic adenosine monophosphate. As a consequence, UGT2B7 depression mediated by LCA can affect its selective catalysis ability to morphine, that may be responsible to acute or chronic morphine tolerance alleviation. These findings might assist to modify antinociception of morphine in clinic.

## Introduction

Bile acids deposited in livers of patients mainly occurs in primary biliary cirrhosis (PBC), and accompanies with many complications, such as hepatic failure (Vlahcevic et al., 1981; Ishibashi et al., 2011; Jankowska and Socha, 2012). As one of vital secondary bile acids derived from primary chenodeoxycholic (CDCA) (de Paiva et al., 2015), lithocholic acid (LCA) is regarded as a main index to diagnose hepatic diseases (Lucangioli et al., 2009) which can be increased significantly in PBC induced extrahepatic cholestasis (Ceryak et al., 1998). When LCA accumulates in liver, the drastic pains (like abdominal pain) occur immediately (Laurin et al., 1994). The intensity of pains are caused by acute or chronic maladies velocity of bile acid (Saraswat et al., 2004; Traub et al., 2008). To better control and release the pain, some opioid analgesic drugs were preferably used in clinic practice. However, accompanying with their applications, tolerance would come into being.

Some serious changes of biomolecules in signal pathways will be acted out subsequently in tolerance. For example, μ-opioid receptor (MOR) will be internalized with sensitivity loss (Werling et al., 1989; Ravindranathan et al., 2009) and auto-phosphorylated with itself expression decline (Deng et al., 2001). Besides, under such condition, calcium flow excreted from the cell membranes will restrain the voltage-dependent Ca^2+^ channels (VDCCs) to remit (Yokoyama et al., 2004). As one of key calcium ions secretory proteins, Ca^2+^/calmodulin-dependent protein kinase IIα (CaMKIIα) which is correlated with opioids tolerance in central nervous system will be suppressed in direction alteration of calcium ion flow (Chen et al., 2010; Hu et al., 2015). Some studies demonstrated that the tolerance induced Ca^2+^ influx can also stimulate *N*-methyl-D-aspartate (NMDA) receptors and cause CaMKIIα auto-phosphorylation at position Thr286 (Fukunaga et al., 1992; Strack et al., 2000), which means Thr286 may be responsible to the reverse of tolerance.

Analgesic opioid drug, morphine as a UGT2B7 substrate, can be converted to morphine-3-glucuronide (M3G) and morphine-6-glucuronide (M6G) in the human livers (Stone et al., 2003; De Gregori et al., 2012). They are playing different roles in morphine antinociceptive tolerance. Compared to morphine and M3G, M6G is more potent opioid agonist which has higher affinity to μ and κ receptors (Frances et al., 1992). It also holds stronger tolerance than morphine when they were treated to mice in the same dose (Faura et al., 1996). In contrast, M3G which owns a weaker μ receptors activation ability was considered to antagonize the analgesia activity of morphine and M6G (Christrup, 1997; Faura et al., 1997). Therefore, morphine induced tolerance would be released by M3G.

To selectively change glucuronidation of morphine, a hypothesis of regulating the enzymatic activity of UGT2B7 was proposed. It was reported that Src (one of non-receptor tyrosine kinase proteins) dependent phosphorylation of UGT2B7 can selectively metabolize catechol-estrogens and 17β-estradiol in COS-1 cells (Mitra et al., 2009, 2011). That may exemplify the hypothesis. More importantly, UGT2B7 downregulation was explored after LCA accumulation in human colon cells, Caco-2 (Lu et al., 2005). It indicated that as an endogenous compound, LCA may potentially repress UGT2B7 in livers and impact its enzymatic activity to metabolize morphine. In order to verify these, we performed several assays *in vitro* and *in vivo*.

## Materials and Methods

### Experimental Materials

Penicillin, streptomycin, fetal bovine serum (FBS), trypsin, Dulbecco’s modified Eagle medium (DMEM) were acquired from GIBCO (Invitrogen Life Technologies, USA). RIPA Lysis Buffer, PMSF were bought from Beyotime (Beyotime Biotechnology, Nantong, China). Goat serum (Santa Cruz, Los Angeles, CA, USA). RNA simple Total RNA Kit (Tiangen, China). Hematoxylin (Sigma, Sigma-Aldrich, Shanghai, China). siRNAs and all the primers for qPCR were designed and synthesized by Sangon Company (Sangon Biotech, Shanghai, China). PrimeScript RT reagent Kit (Perfect Real Time) and SYBR Premix Ex Taq (Tli RNaseH Plus) were purchased from Takara Company (Takara, Japan). Bull Serum Albumin (BSA), RIPA, PMSF were obtained from Beyotime Company (Beyotime Biotech, Shanghai, China). Rabbit polyclonal antibody against μ-opioid receptor (MOR) (ab10275), CaMKII (ab22609), Thr286 phosphorylation of CaMKII (ab32678) and UGT2B7 (ab126269) were purchased from the Abcam Company (Shanghai, China). Mouse monoclonal antibody against glyceraldehyde phosphate dehydrogenase (GAPDH) was purchased from KangChen Bio-tech Inc. (Shanghai, China). HRP-conjugated goat anti-rabbit or anti-mouse IgG(G+L) was obtained from MultiSciences Biotech Co, Ltd. (Hangzhou, China). ECL Western Blotting Substrate (Biomiga, San Diego, CA, USA). Lipofectamine 3000 and 2-Solution DAB Kit was purchased from Invitrogen Life Technologies Company (San Jose, CA, USA). Mouse cAMP Parameter Assay ELISA Kit was purchased from R&D systems (Minneapolis, MN, USA).

Lithocholic acid was purchased from the Sigma-Aldrich Company (St. Louis, MO, USA). Morphine, M3G, and M6G were purchased from Cerilliant Corporation (Round Rock, TX, USA). M6G-d3, as internal standard, was labeled by Biomag System Company (Changshu, Jiangsu, China). HPLC-grade methanol and formic acid were purchased from Tedia Company (Fairfield, OH, USA). Ultrapure water (18.2 MΩ) was obtained from an ELGA–pure lab Ultra system (High Wycombe, UK).

Waters HLB C18 solid phase extraction (SPE) columns were obtained from Waters Company (Waters, Milford, MA, USA). Agilent HILC PLUS SB-C18 column (2.1 mm × 50 mm, 3.5 μm, Agilent, USA). Data acquisition and processing were performed using Analyst 1.5.2 (AB SCIEX). All centrifugations were performed on an Eppendorf 5415R Refrigerated Micro-centrifuge (Eppendorf, Germany).

### Cell Cultivation

Normal human liver cells, HL-7702 were cultured at an atmosphere of 5% CO_2_ and 95% air at 37°C. DMEM, being as its cultivation medium supplemented with 10% FBS, 1% penicillin, streptomycin. The cells were passaged by 0.25% EDTA mixed with trypsin. All the compounds diluted with DMSO were added into DMEM medium and incubated for a serious of hours. Then we changed old medium to fresh one containing with different concentrations of each compound every 24 h. Same volumes of DMSO (less than 0.1%) were treated the cells as control.

### Animals

ICR mice (male, body weight of 20–25 g) were obtained from Experimental Animals Department of Zhejiang University. Animals were fed in a breeding room with temperature at 25°C, humidity of 50 ± 10%, and a 12 h dark–light cycle. They were free to approach water and rodent chows all the time. All the experimental animals were cultivated under the above conditions for 7 days to acclimate, then they were weighted and recorded every day before the drugs treatment. The animals were administrated with different compounds twice a day based on the mice weights. Animal experiments were strictly conducted in accordance with the protocols approved by the Ethics Committee for Animal Studies at Zhejiang University. The paperwork was according to the documentation of ‘The Detailed Rules and Regulations of Medical Animal Experiments Administration and Implementation’ (Document No. 1998–55, Ministry of Public Health, China).

### siRNA Transfection

HL-7702 cells were seeded in 6-well plates at a density of 0.5 × 10^5^/well. The pairs of sequences for siRNA-621 which can specific knocked down homo UGT2B7 were CCUACGUACCUGUUGUUAUTT as positive sense, and AUAACAACAGGUACGUAGGTT as antisense, respectively. The negative control (NC) siRNA was with sequences of UUCUUCGAACGUGUCACGUTT as positive sense and ACGUGACACGUUCGGAGAATT as antisense, respectively. In brief, 200 nM siRNA was treated in non-serum DMEM medium combining with the reagent of Lipofectamine 3000 and incubated 5 min at room temperature, after that all the mixture solutions were added to the cells. After 5.5 h incubation, the medium was mixed with serum including all the compounds and cultivated for another 48 h (transfer the medium again at 24 h). Then all the cells were harvested to make a further detection.

### Quantitative Real-Time PCR

HL-7702 cells were seeded in 6-well plates at a density of 0.5 × 10^5^/well. After administrated with different concentrations of compounds, the cells were cultivated for 48 h (changed the medium including drugs at 24 h). Then total RNAs were isolated from cells, and cDNA was synthesized. Real-time PCR was carried out on a StepOne plus Real-Time PCR system, samples were mixed by SYBR. Expression of each target mRNA was normalized to housekeeping gene glyceraldehyde-3-phosphate dehydrogenase (GAPDH). The primer pairs for related genes were based on the **Table 1**.

**Table 1:** The information of primer sequences used in qPCR assay.

Names	ID	Sense (5′–3′)	Antisense (5′–3′)
hUGT2B7	NM_001074.2	AAGAAAGGGCCAACGTAATT	AGAGCCGAGTATTGAGACCTAA (108 bp)
hMOR	NM_000914.4	ACAGGCAAGGTTCCATAGATTG	CTGGCATAATGAAGGCGAAGAT (105 bp)
hCaMKIIα	NM_015981.3	ATCCCCACATCCACCTGAT	GGGTGATGACATGGGAGAAT(304 bp) (Cavazzin et al., 2004)
hGAPDH	NM_002046.5	CATGAGAAGTATGACAACAGCCT	AGTCCTTCCACGATACCAAAGT (113 bp)
musMOR	AH_005396.2	GACTGCTCTGACCCCTTAGCTCC	TTGCCATCAACGTGGGACAAG (80 bp)
musCaMKIIα	NM_009792.3	AAACAAGAAGAACGATGGTGTGAAG	GTGTTGGTGCTCTCAGAAGATTCC (81 bp)
musGAPDH	NM_001289726.1	GAGAAACCTGCCAAGTATGATGAC	AGAGTGGGAGTTGCTGTTGAAG (129 bp)

### Tests of Acute and Chronic Morphine Tolerance and Antinociception in Mice

ICR mice were pretreated with LCA (p.o.) for 7 days at different concentrations (10 mg/kg, 50 mg/kg, and 100 mg/kg) in 0.9% saline solutions. Then the acute or chronic morphine tolerance mice models were established and validated based on the method previously reported (Hu et al., 2015). During this period, each group of mice was treated with different doses of LCA and same volumes of 0.9% saline solutions as a control. Tail-flick assay was performed to estimate basal nociception and morphine-induced antinociception as described previously (Tang et al., 2006; Yang et al., 2011). The latency of quick tail-flick response was recorded and expressed as the percentage of maximal possible effect (MPE%) based on the following formula:

$$\%MPE\;=\frac{100\;\times \;(postdruglatency\;-\;predruglatency)}{\left(cutoff\;-\;predruglatency\right)}$$

A cutoff time of 12 s was used to prevent from the potential injure of mice tissues.

### Immunohistochemistry

The livers of mice were resected and harvested, then fixed in 4% paraformaldehyde for 12 h and kept in cold 30% sucrose-PBS solution overnight. The sections were exposed to 0.3% H_2_O_2_ (final concentration in PBS) to reduce the endogenous peroxidase for 30 min. Then, the reaction was blocked with 10% goat serum in PBS, and the reaction mixture incubated with primary rabbit polyclonal antibodies UGT2B7 (diluted with 1:500) overnight at 4°C and HRP-conjugated goat anti-rabbit polyclonal secondary antibody (1:2000) for 1 h. All sections were treated with a peroxidase substrate solution. Hematoxylin and 3,3′-diaminobenzidine tetra hydrochloride (DAB) were used as counterstaining reagents. The prepared slides were observed with ×200 of magnification under Olympus BX41 microscope to measure the UGT2B7 expression. The NC group without primary antibody was carried out with the same procedure as described above.

### Western Blotting Assay

The total proteins were extracted from the cell lysates or mouse brain prefrontal cortexes (mPFCs) after they were incubated with RIPA lysis buffer containing 1x PMSF. The protein concentration of each sample was determined by BCA kit. Then, 50 μg protein samples were subjected to SDS-PAGE and transferred on PVDF membranes. The membranes were incubated in blocking solution containing 5% BSA in TBST buffer (100 mM Tris-HCl, pH7.4, 150 mM NaCl and 0.1% Tween 20) for 1 h at room temperature before overnight incubated with primary antibody at 4°C. The primary antibodies were diluted by TBST buffer including UGT2B7 (1:1000), MOR (1:500), CaMKIIα (1:5000) and Thr286 phosphorylation of CaMKIIα (1:2000). After washing three times to remove the primary antibody, the HRP-conjugated goat anti-rabbit IgG (1:2000) as secondary antibody was used for detection by ECL substrates. Target proteins were visualized by exposing the membranes to G-box chemiluminescence apparatus carried Genesnap system for 5 min exposure. GAPDH was paralleled by using as an internal reference to normalize the expression of UGT2B7, MOR, CaMKIIα, and Thr286 phosphorylation of CaMKIIα. Each integrated optical density (IOD) value of stripe was measured and calculated by Image-Pro Plus 6.0 Software (Media Cybernetics Corporate, Bethesda, MD, USA).

### Preparation of Mice Liver Samples Using SPE

The integral livers of mice were extracted, then homogenized by Automatic Tissue Grinder by 15 ml purified water included with 0.5% formic acid, then centrifuged for 10 min at 4°C and 3,000 × g. After that, 2 ml of acetonitrile was add to 1 ml supernatant to precipitate proteins with centrifuging at 13,000 × g for 10 min. M6G-d3 (IS) was applied as internal standard and added to the supernatant with a final concentration of 10 ng/ml in each sample. The SPE process based on the method previously reported (Yang et al., 2016). In brief, the mixture was loaded onto balanced HLB Waters SPE column to process a secondary pretreatment activated by 5 ml methanol and eluted by 1 ml acetonitrile. The eluate, then, was evaporated and dried in a centrifugal thickener. After dissolved in 100 μl mobile phase and centrifuged, 10 μl supernatant was injected into HPLC–MS/MS to determine morphine and its metabolites. The concentrations of analytes were reflected the quantity in 1ml mice liver homogenates.

### HPLC–MS/MS Methods Validation

All the mice liver homogenate samples were determined by HPLC–MS/MS. Agilent 1290 infinity LC system equipped with a G4220A quaternary pump, G4226A auto sampler and G1330B 1290 thermostat. AB SCIEX 4000 plus triple quadrupole mass spectrometer (AB SCIEX Technologies) with an electrospray ionization source were performed for the HPLC–MS/MS analysis. The auto-sampler was maintained at 4°C, and the temperature for column compartment was set at 30°C. Chromatographic separations were achieved on an Agilent HILC PLUS SB-C18 column (2.1 mm × 50 mm, 3.5 μm). The mobile phase for analyzing morphine, M3G, M6G, M6G-d3 (ISTD) consisted of 0.05% formic acid in purified water (A) and methanol (B) with a gradient elution of 95% A at 0–1 min, 98% B at 1–3 min, 98% B at 3–6 min, 95% A at 6–7 min with a flowing rate of 0.25 ml/min.

Mass spectrometer with ESI source was operated in positive ionization mode. Parameters of which were set as following: collision energy, 36 eV for morphine and 43 eV for M3G, M6G, M6G-d3 (ISTD); Declustering potential, 89 V for morphine, and 85 V for metabolites and M6G-d3(IS). The integral temperature of drying gas, 350°C; drying gas flow, 8 L/min; temperature for ionization 550°C. Data were acquired using the Analyst 1.5.2 (AB SCIEX) in the multiple reaction monitoring (MRM) mode by recording ion currents for the following transitions: 286–200.9 m/z for morphine, 462.1–286.1 m/z for M3G and M6G, 465.1–289.1 m/z for M6G-d3 (ISTD) in positive mode. The method has been validated.

### ELISA Assay

The ELISA assay was performed to determine the synthesis concentrations of cyclic adenosine monophosphate (cAMP) in mPFCs by kit. In brief, cold 0.1 M HCl was added into the tissue homogenates of mPFCs at a 5:1 ratio, after centrifuged, the supernatants and standard samples were prepared following the instructions of the kit. The sample concentrations were determined at 450 nm on a microplate reader and calculated by standard curve.

### Data Analysis

Statistics data were expressed as mean ± SEM derived from three paralleled independent experiments. Unpaired two-side Student’s *t*-test was applied for comparisons of two groups’ data. A *P*-value <0.05 was considered statistically significant. The immunohistochemistry assay and western blotting assays were measured by IOD values or the ratios of IOD values to summary of areas by Image Pro Plus 6.0 software. Western blotting assay was then normalized to the IOD value of GAPDH in each group. The figures and statistics were all counted by GraphPad Prism 5.0 (GraphPad Software Inc., San Diego, CA, USA).

## Results

### LCA Mediated UGT2B7 Down-Regulation in Morphine Tolerance-Like HL-7702 Cells

**FIGURE 1:**
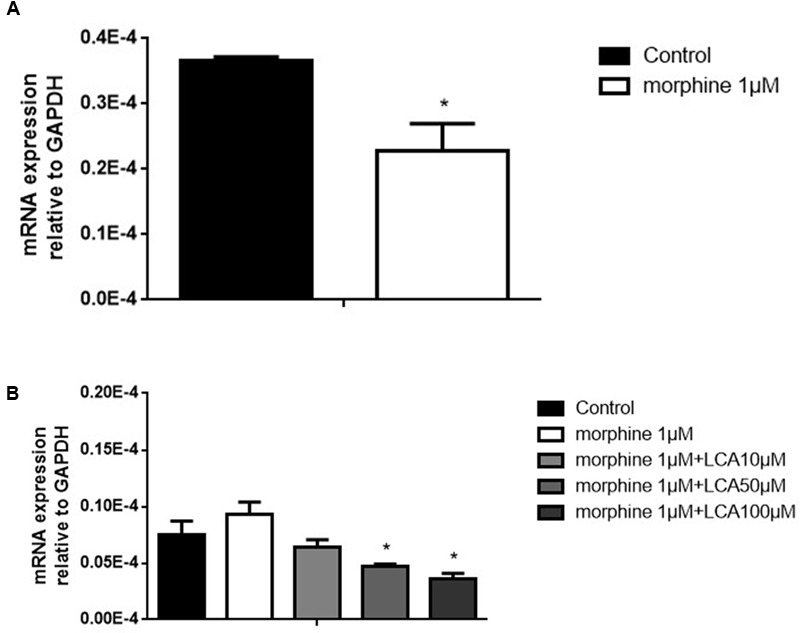
**The effect of LCA on UGT2B7 expression in morphine tolerance-like HL-7702 cells. (A)** MOR expressed in the cells treated with 1 μM morphine for 48 h to determine the generation of tolerance. **(B)** UGT2B7 expressed in the cells co-incubated with 1 μM morphine and various concentrations of LCA (10, 50, 100 μM) for 48 h before same doses of LCA 48 h pretreatment in each group. Results were normalized to GAPDH with mean ± SEM of three independent experiments. DMSO was used as control in each group. Pairwise comparisons were calculated by student *t*-test to calculate *P*-values (^∗∗∗^*P* < 0.001, ^∗∗^*P* < 0.01, ^∗^*P* < 0.05).

Morphine tolerance-like HL-7702 cell model was established and validated. In brief, 1 μM morphine was added to HL-7702 cells for 48 h, then the total mRNA was extracted from the cells. The results indicated that MOR decreased significantly compared to control group, thus the morphine tolerance-like status was induced in HL-7702 cells (**Figure 1A**). In order to investigate the effect of LCA on UGT2B7 expression, HL-7702 cells were treated with different concentrations of LCA (10, 50, 100 μM) for 48 h, then the cells were co-incubated with 1 μM morphine for another 48 h. From the results, 50 μM and 100 μM of LCA can significantly depress UGT2B7 (**Figure 1B**).

### UGT2B7 Knockdown Regulated MOR and CaMKIIα Expression Increase in Morphine Tolerance-Like HL-7702 Cells

**FIGURE 2:**
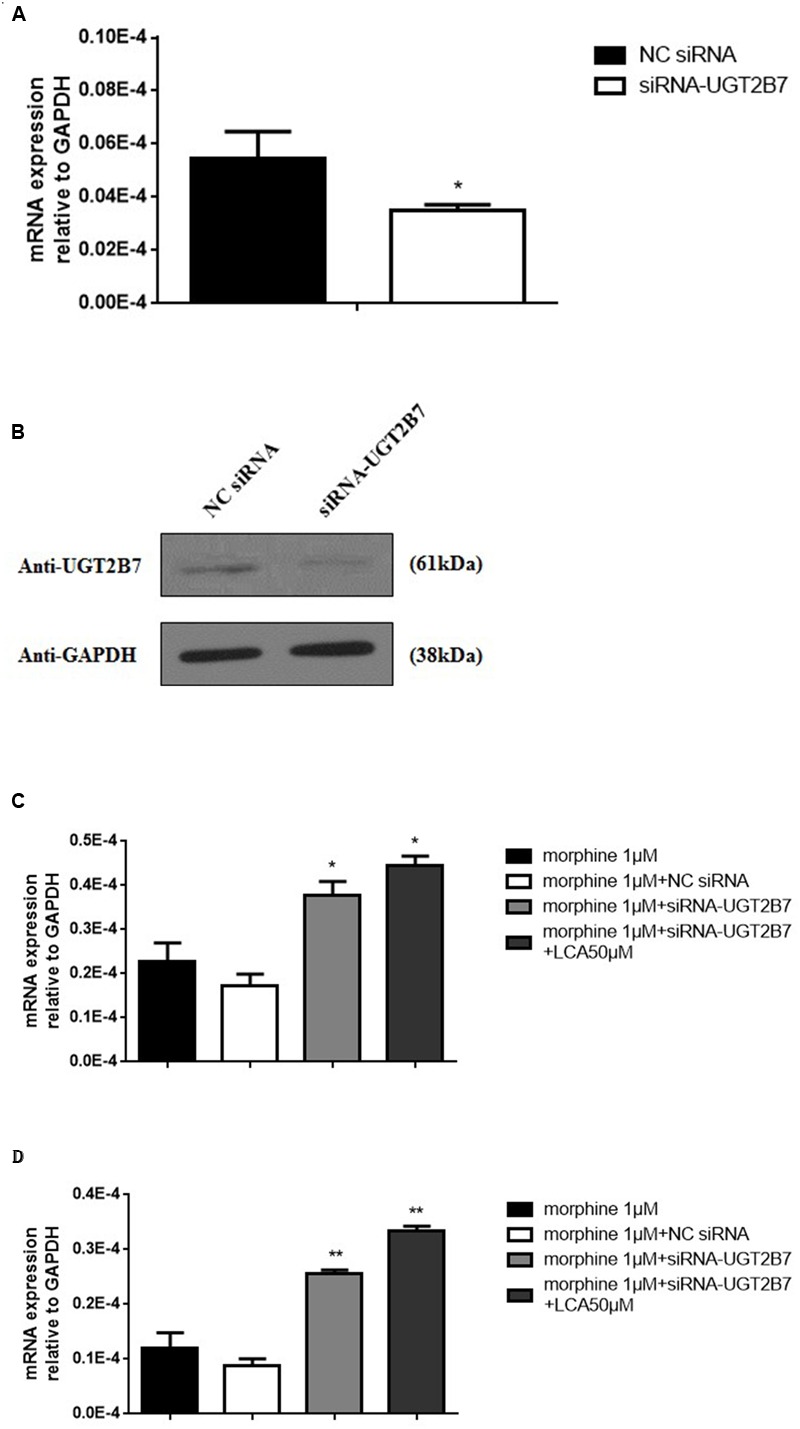
**Alteration Analysis of genes correlated to morphine tolerance after UGT2B7 knocked down in morphine tolerance-like HL-7702 cells. (A)** UGT2B7 mRNA expression or **(B)** protein expression was determined in the cells which transfected with siRNA (UGT2B7). Tolerance related genes of **(C)** MOR or **(D)** CaMKIIα mRNA expression was determined in siRNA(UGT2B7) transfection cells pretreated with 50 μM LCA for 48 h then co-incubated with 1 μM morphine for additional 48 h. Results were normalized to GAPDH with mean ± SEM of three independent experiments. DMSO or NC RNA were used as control. Pairwise comparisons were calculated by student *t*-test to calculate *P*-values (^∗∗^*P* < 0.01, ^∗^*P* < 0.05).

To further research whether UGT2B7 involved in morphine tolerance, we transfected siRNA to specifically knock down UGT2B7 in HL-7702 cells. The data revealed that UGT2B7 was dominantly inhibited in both levels of mRNA and protein expressions compared to NC siRNA transfection (**Figures 2A,B**). Meanwhile, MOR (**Figure 2C**) and CaMKIIα (**Figure 2D**) were all enhanced in HL-7702 cells exposed to 1 μM morphine. However, MOR and CaMKIIα sequentially increased after the cells were pretreated with 50 μM of LCA (**Figures 2C,D**).

### Alleviation of Acute or Chronic Morphine Tolerance *via* Inhibiting UGT2B7 in Mice Liver by LCA

**FIGURE 3:**
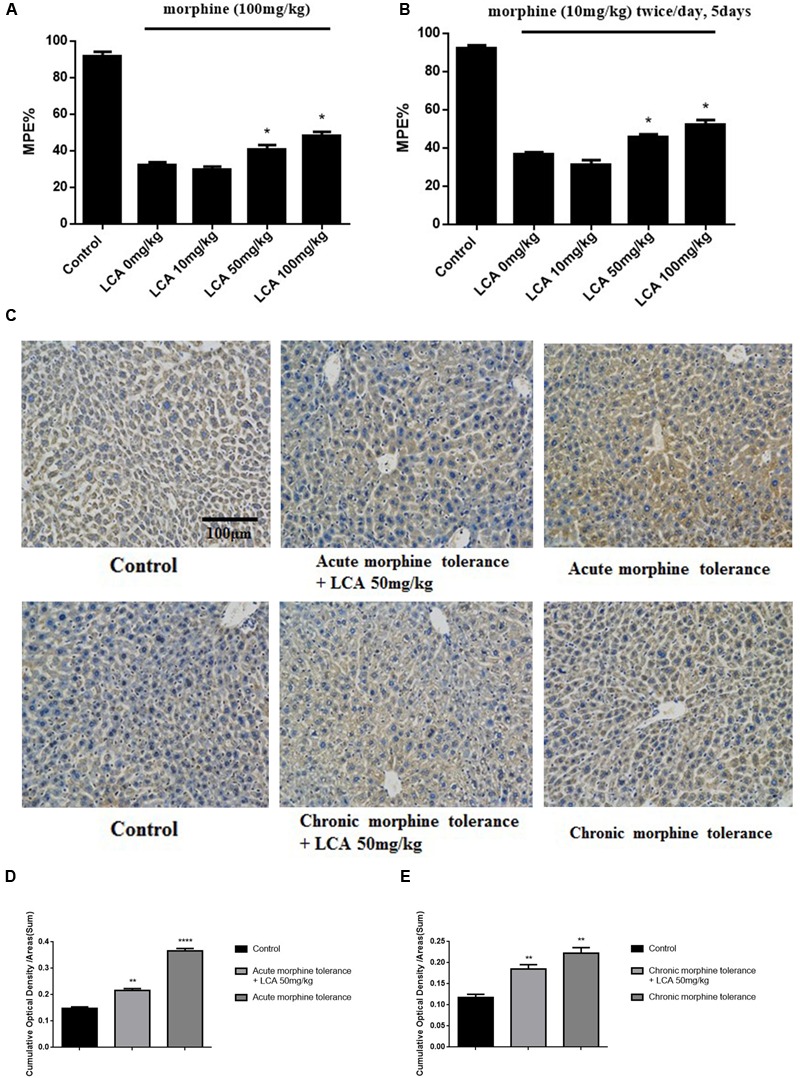
**The effect of LCA on acute or chronic morphine tolerance mice.** Tail-flick assays were performed to **(A)** acute or **(B)** chronic morphine administration mice treated with different concentrations of LCA (10, 50, 100 mg/kg; p.o.) based on our previous method for evaluating the changes of morphine tolerance. Then maximum possible effect (MPE%) were counted based on the formula above. **(C)** Immunohistochemistry assay was developed to clearly investigate UGT2B7 expression in mice livers at magnification of 200×. The ratios of IOD values to summary areas were measured and counted to reflect the integral UGT2B7 (labeled with brown color) variations in **(D)** acute or **(E)** chronic morphine tolerance mice livers by Image Pro Plus 6.0 software. Results were with mean ± SEM of three independent experiments. Saline was used as control in each group of mice. Pairwise comparisons were calculated by student *t*-test to calculate *P*-values (^∗∗∗∗^*P* < 0.0001, ^∗∗^*P* < 0.01, ^∗^*P* < 0.05).

To explore the functions of LCA *in vivo*, morphine was injected into mice to acquire acute or chronic tolerance status based on the methods reported previously (Hu et al., 2015). Significant reverse of morphine tolerance was reflected from MPE% values after acute or chronic mice accepted LCA administration. Specifically, 50 mg/kg or 100 mg/kg of LCA can attenuate morphine antinociceptive tolerance *via* increasing their MPE% values by 26.4 or 49.4% for acute tolerance and 24.3 or 42.5% for chronic tolerance. However, 10 mg/kg of LCA administration exhibited no significant difference no matter in acute or chronic morphine tolerance groups (**Figures 3A,B**). Besides, immunohistochemistry assay was performed in mice livers to further detect UGT2B7 expression under 50 mg/kg of LCA regulation. The ratios of IOD and summary areas were measured by Image Pro Plus 6.0 software. From the results, UGT2B7 expression increased in the mice livers under acute or chronic morphine tolerance. In contrast, UGT2B7 decreased in the mice livers when they were exposed to LCA (**Figures 3C,D,E**).

### LCA Induced Accumulation Variants of Two Metabolites in Acute or Chronic Morphine Tolerance Mice Livers

**FIGURE 4:**
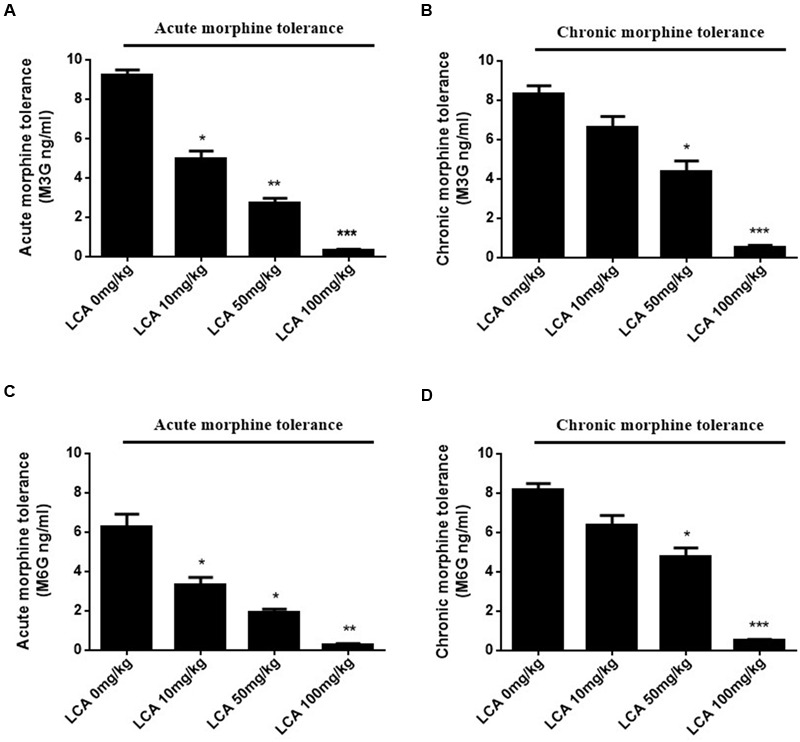
**Concentrations of M3G and M6G in different doses of LCA (10, 50, 100 mg/kg; p.o.) treated acute or chronic morphine tolerance mice livers were determined by (SPE)–HPLC–MS/MS based on the method shown above.** Concentration changes of M3G and M6G were measured in **(A,C)** acute or **(B,D)** chronic morphine tolerance mice liver homogenates. 0 mg/kg LCA reflects saline treatment as control in each group of mice. Results were with mean ± SEM of three independent experiments. Pairwise comparisons were calculated by student *t*-test to calculate *P*-values (^∗∗∗^*P* < 0.001, ^∗∗^*P* < 0.01, ^∗^*P* < 0.05).

To illustrate whether LCA can regulate enzymatic activity of UGT2B7. Morphine, M3G, and M6G were determined in acute or chronic tolerance morphine mice livers with liver LCA accumulation. The results indicated that the concentrations of M3G (**Figures 4A,B**) and M6G (**Figures 4C,D**) were all declined. In addition, both of the ratios of M6G to morphine and M6G to M3G experienced downtrend. It was important to mention that no matter in acute or chronic morphine tolerant mice, 100 mg/kg of LCA resulted in the lowest ratios (**Table 2**).

**Table 2:** Concentration ratios of M6G to morphine or M6G to M3G in LCA (10, 50, 100 mg/kg, p.o.) treated acute or chronic morphine tolerance mice livers.

Group	Acute morphine tolerance		Chronic morphine tolerance	
	[M6G/Morphine]	[M6G/M3G]	[M6G/Morphine]	[M6G/M3G]
LCA 0 mg/kg	1.30 ± 0.15	1.53 ± 0.08	1.06 ± 0.11	1.02 ± 0.12
LCA 10 mg/kg	1.20 ± 0.26	1.51 ± 0.27	0.79 ± 0.16^∗^	1.03 ± 0.22
LCA 50 mg/kg	1.09 ± 0.30^∗^	1.43 ± 0.30	0.64 ± 0.19^∗∗^	0.91 ± 0.29
LCA 100 mg/kg	0.83 ± 0.28^∗∗^	1.10 ± 0.45 ^∗^	0.51 ± 0.27^∗∗∗^	0.77 ± 0.51^∗∗^

*Drug administration method was shown above. 0 mg/kg LCA reflects saline treatment as control in each group of mice. Results were with mean ± SEM of three independent experiments. Pairwise comparisons were calculated by student *t*-test to calculate *P*-values (^∗∗∗^*P* < 0.001, ^∗∗^*P* < 0.01, ^∗^*P* < 0.05).*

### Analysis of cAMP Concentration in Acute or Chronic Morphine Tolerance Mice Prefrontal Cortexes

**FIGURE 5:**
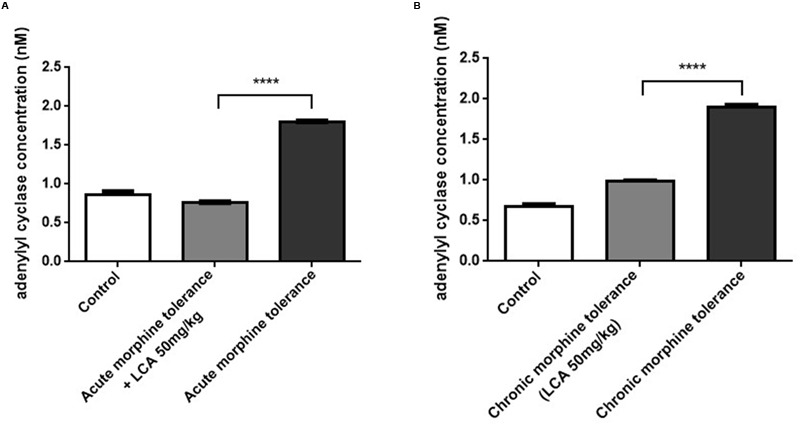
**The level of cAMP synthesis in mPFCs.** Concentrations of cAMP determined by ELISA kit in *(A)* acute or *(B)* chronic morphine tolerance mice treated with LCA 50 mg/kg based on the method shown above. Results were with mean ± SEM of three independent experiments. Saline was used as control in each group of mice. Pairwise comparisons were calculated by student *t*-test to calculate *P*-values (^∗∗∗∗^*P* < 0.0001).

cAMP, as one of vital intracellular signaling molecules can be used to estimate the phosphorylation alterations of total proteins in targeted animal tissues. To estimate whether LCA contributes to auto-phosphorylation changes of proteins correlated to tolerance in mPFCs, the cAMP concentrations were quantified by ELISA kit. From the data, cAMP in acute or chronic morphine tolerance mice treated with 50 mg/kg of LCA was changed from 1.80 to 0.76 nM or 1.90 to 0.98 nM, respectively, compared to no LCA treatment (**Figures 5A,B**). It indicated, protein phosphorylation had taken place in these brain sections.

### LCA Regulation of Proteins Correlated to Tolerance in Mice Prefrontal Cortexes

**FIGURE 6:**
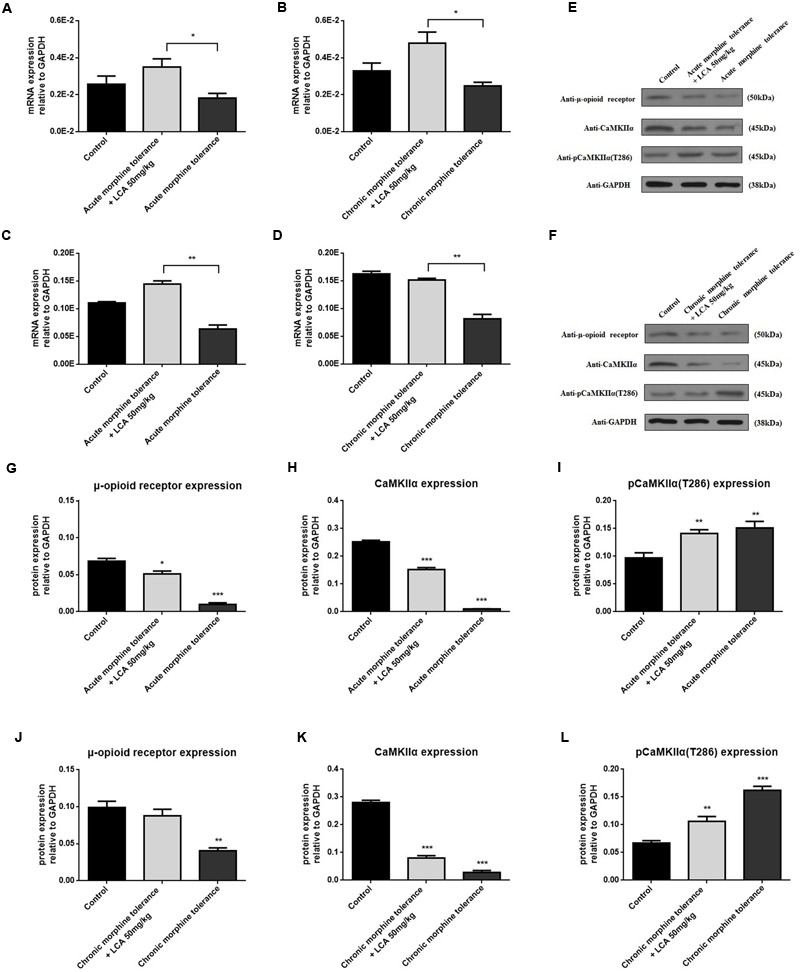
**LCA mediated mRNA and protein expression changes of MOR and CaMKIIα in mPFCs after the mice suffered with acute or chronic morphine induced tolerance.** mRNA expression changes of **(A,B)** MOR or **(C,D)** CaMKIIα was determined in mPFCs after the mice treated with 50 mg/kg LCA under **(A,C)** acute or **(B,D)** chronic morphine tolerance. Protein expression including MOR, CaMKIIα, and auto-phosphorylation of CaMKIIα at Thr286 was measured in mPFCs after the mice treated with 50 mg/kg LCA under **(E)** acute or **(F)** chronic morphine. IOD value of each protein including MOR, CaMKIIα, and auto-phosphorylation of CaMKIIα at Thr286 was normalized to GAPDH under **(G–I)** acute or **(J–L)** chronic morphine tolerance. Results were with mean ± SEM of three independent experiments. Pairwise comparisons were calculated by student *t*-test to calculate *P*-values (^∗^*P* < 0.05).

To determine the alterations of some proteins correlated to tolerance, acute or chronic morphine tolerant mice were treated with 50 mg/kg of LCA. The results showed that mRNA and protein of MOR increased predominantly in mPFCs (**Figures 6A,B,E–G,J**). We found, compared to MOR, another vital protein, CaMKIIα had a similar uptrend (**Figures 6C–F,H,K**). In contrast, auto-phosphorylation of CaMKIIα at Thr286 was reduced in acute or chronic morphine tolerance mice treated with 50 mg/kg of LCA (**Figures 6E,F,I,J**). This result was in accordance with the concentration alterations of cAMP in mPFCs.

## Discussion

**FIGURE 7:**
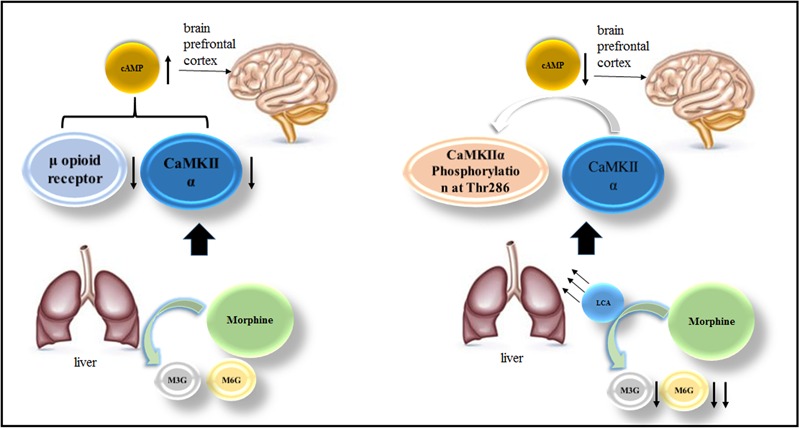
**The mechanism of LCA mediated UGT2B7 down-regulation and acute or chronic morphine tolerance reverse**.

In this study, HL-7702 cells and acute or chronic morphine tolerance mice models were applied to investigate LCA mediated alterations of UGT2B7 expression and enzymatic activity. At the meantime, we also explored the changes of some signals or proteins correlated to morphine tolerance in the mice brains under LCA regulation. The brief mechanism has been illustrated in the diagram of our research (**Figure 7**).

It was reported that MOR can be measured as a vital index to evaluate the tolerance status (He et al., 2009). Its expression can be significantly reduced in SH-SY5Y cells treated with 1 μM morphine for 24 and 48 h (He et al., 2010). To demonstrate whether LCA can regulate UGT2B7 under tolerance status *in vitro*. We established the morphine tolerance-like cells of HL-7702 and found MOR was significantly repressed in the cells, which means tolerance-like models had been established.

Yuan et al. once reported that 10 μM of LCA can downregulate UGT2B7 in Caco-2 cells significantly. In this research, we found morphine can induce UGT2B7 in HL-7702 cells though it was not remarkable compared to LCA. UGT2B7 was also upregulated in mice livers in acute or chronic morphine-induced tolerance. The above results indicated that morphine would be a potential weak agonist to UGT2B7. Similarly, UGT2B7 can be regulated by its substrate zidovudine though this induction performance may be distinct in different cell lines. Zidovudine at the concentration from 2 to 100 μM can activate UGT2B7 in human liver carcinoma cell line (HepG2), in contrast, it showed a downtrend from 50 to 2500 μM in human thyroid carcinoma cell (THLE2) (Fang et al., 2014). Therefore, further research should be developed whether morphine has the similar effects like zidovudine when it regulates UGT2B7.

To clarify whether LCA intervenes the enzymatic activity of UGT2B7 by blocking its expression, each concentration of morphine, M3G or M6G was determined in the livers of acute or chronic morphine-induced tolerance mice treated with multiple concentrations of LCA. The results showed that both concentrations of M3G and M6G were continuously reduced with the increase of it. Similar trend was observed in each concentration ratio of M6G to morphine or M6G to M3G which elucidated that M6G concentration decreased more rapidly than the other two compounds. That is to say, regio-selective glucuronidation caused by UGT2B7 downregulation could change the enzymatic activity and decrease the morphine tolerance.

In this experiment, we extracted mPFCs to investigate tolerance dependent signal variations. PFC is a main region in brains to study opioids-induced tolerance, withdrawal and addiction (Contet et al., 2008; Liu et al., 2015; Peregud et al., 2015). From our results, cAMP synthesis decreased significantly after mice were pretreated with 50 mg/kg of LCA compared to control groups, which showed the protein targets correlated to tolerance underwent dephosphorylation. By that means, we found phosphorylation of CaMKIIα at Thr286 was restricted in the tolerance induced mPFCs, in contrast, CaMKIIα was prompted. Since Ca^2+^ influx mediated the motivation of the N-methyl-D-aspartate (NMDA) receptor may lead to CaMKIIα auto-phosphorylation at Thr286 and induce full activation of the kinase in morphine-induced tolerance (Strack et al., 2000; Zhao and Joo, 2006). Thus, LCA deposited in the mice livers might affect morphine brain distribution and attribute to CaMKIIα dephosphorylation at Thr286 site.

In summary, LCA mediated UGT2B7 down-regulation can repress the auto-phosphorylation of CaMKIIα and increase cAMP contents in mPFCs. The expression of UGT2B7 may change its enzymatic activity by regio-selective altering the glucuronidation of morphine metabolites in mice livers reflected from the decrease ratio of M6G to M3G. This mechanism might be guided to prevent morphine antinociceptive tolerance in clinic.

## Author Contributions

Conceived and designed the experiments: ZY, LL, HH, MX, LY, ZW, and SZ. Performed the experiments: ZY and LL. Analyzed the data: ZY and LL. Wrote or contributed to the writing of the manuscript: ZY, LY, and SZ. Contributed reagents/materials/analysis tools: JG.

## Conflict of Interest Statement

The authors declare that the research was conducted in the absence of any commercial or financial relationships that could be construed as a potential conflict of interest.
